# Influence of Nanofibrillated Bacterial Cellulose on the Properties of Ordinary and Expansive Mortars

**DOI:** 10.3390/ma15062094

**Published:** 2022-03-11

**Authors:** Emika Kuroiwa, Nguyen Xuan Quy, Yukio Hama

**Affiliations:** 1 Division of Sustainable and Environmental Engineering, Muroran Institute of Technology, Muroran 050-8585, Japan; 20041026@mmm.muroran-it.ac.jp; 2 Faculty of Civil Engineering, Hanoi Architectural University, Hanoi 100000, Vietnam; egcmat@gmail.com; 3 Graduate School of Engineering, College of Environmental Technology, Muroran Institute of Technology, Muroran 050-8585, Japan

**Keywords:** nanofibrillated bacterial cellulose, setting time, compressive strength, frost resistance, expansive additive, curing conditions

## Abstract

This study uses two types of nanofibrillated bacterial cellulose (NFBC), a culture solution containing NFBC (B_f_) and a purified solution (P_f_), to investigate the influence of NFBC on the basic properties of mortar. The flow test, air content test, setting time test, restraint expansion test, dry shrinkage test, strength test and freeze–thaw test were performed. The results show that the flow of fresh mortar increases for B_f_ and decreases for P_f_, while the setting time of mortar is delayed for B_f_. The dry shrinkage is slightly decreased as a result of using NFBC in expansive mortar. In addition, for both types of NFBC, the strength is not significantly affected in ordinary mortar, while the compressive strength tends to increase slightly after 28 days of underwater curing in expansive mortar. Moreover, the frost resistance improves as the air content increases in ordinary mortar. In expansive mortar, the frost resistance is improved for B_f_, but the frost resistance is not improved for P_f_. This investigation has revealed that NFBC can be used as an admixture to improve the properties of mortar, such as frost resistance.

## 1. Introduction

Cellulose is the most important biomass resource and accounts for one third of natural plant matter [1]. If some of the materials and energy derived from fossil resources can be replaced with renewable carbon-neutral biomass, it will lead to the solution of global environmental and resource problems. Therefore, research on materials using cellulose as a part of raw materials is necessary [2].

Cellulose nanofiber (CNF) is a typical example of a cellulose material. CNFs are cellulose fibers with nanometer-scale diameters. They have received much attention in recent years as next-generation large-scale industrial materials and green nanomaterials, thanks to their light weight, high strength, and low thermal-expansion rate [3,4,5]. Research into how to manufacture and use CNFs in applications has been ongoing since the early 2000 s. CNFs with various fiber widths, lengths, and properties have been used in a wide range of fields [6,7]. Taking advantage of these features, CNF is being used as a reinforcing material to increase the strength of plastics [7]. CNF obtained by mechanically defibrating or chemically treating wood pulp has fiber widths of approximately 4 to 20 nm, while CNFs made from agricultural waste such as sugar cane and citrus pomace have fiber widths of approximately 20 to 50 nm [3]. Nanofibrillated bacterial cellulose (NFBC) produced by fermentation using sugar and molasses as raw materials has also been developed [8,9]. NFBC has a relatively uniform fiber width, does not contain lignin or hemicellulose, has a long fiber length, and is very safe, finding applications in foods and pharmaceuticals [1,7].

Some studies have used CNFs made from pulp for cementitious materials. Obinna et al. [10] showed that the flexural strength increased by CNF prepared by mechanical defibrillation of bleached softwood pulp and the flexural strength increased by CNF derived from algae, but the flexural strength decreased by the commercial CNF [11]. Using CNF made from eucalyptus pulp promoted the hydration reaction and improved the compressive strength in cement paste, [12]. Li et al. [13] reported that the flexural and compressive strength of cement paste with 0.15 wt% CNF increased 15% and 20%, respectively, due to the high degree of hydration and dense microstructure. Wataru et al. [14] showed that CNF made from chemical pulp improved the tensile strength of concrete under dry curing. Jose et al. [15] reported that adding CNF to cementitious systems reduced the sulphate penetration. Moreover, Hassan et al. [16] pointed out from the data obtained by X-ray microtomography that the cement paste with CNF may increase the voids and the compressive strength decreased by CNF. For the drying shrinkage of cement paste increase by the addition of large amounts of CNF, [17]. Zhidong et al. [18] reported that the cement hydration is eventually much enhanced in the long term, but the chemical effect of CNF on cement hydration is small in the short term. These facts suggest that CNF affects the properties of cementitious materials, but the results may differ depending on the manufacturing process and other factors [19].

Studies on mixing steel fiber, synthetic fiber, plant fiber, etc., in mortar and concrete as a reinforcing material to improve the tensile strength and crack resistance have been ongoing. Many studies on applying steel fibers and synthetic fibers to concrete have been carried out for a long time, and they are used in various actual structures [20,21]. Among them, various studies used different shapes and dimensions of steel fibers and synthetic fibers made by different materials (polypropylene and polyethylene, polyolefin, polyvinyl alcohol, etc.), and many experiments will be conducted in order to make better structures in the future. It is expected that experimental and theoretical applications will be carried out [22]. For example, various modifications of shape improved fiber-matrix bound and increased efficiency of the fibers, and because the fine fibers are densely in the cement matrix, they control the opening and propagation of microcracks. It has been reported that longer fibers control larger cracks and contribute to increase the final strength of fiber-reinforced cement-based composites [22]. In addition, Pei et al. [23] studied the mixing of various types of fibers into concrete, such as basalt fibers, polypropylene fibers and glass fibers, and their results showed that no matter which fiber was mixed into the concrete, the flexural strength improved and the drying shrinkage decreased. Especially when basalt fiber was mixed, the concrete showed excellent resistance to the dimension-changing rate. On the other hand, it was also shown that the fluidity and compressive strength of concrete decreased as the mixing ratio of fibers increased.

In recent years, further use of plant fibers has been expected for the purpose of reducing the environmental load. For example, Muhammad et al. [24] showed that the flexural strength, energy absorption, and toughness indices of concrete were increased by wheat straw. Another study examined the structural and environmental performance of structures using concrete mixed with bamboo [25]. However, it has been pointed out that fibers made from plants will deteriorate under an alkaline environment, such as cement [24,26,27]. Therefore, the current situation is that plant fibers have not been widely used as a substitute for other fibers.

Fortunately, NFBC treated at pH 12 or higher under high alkaline conditions in the lysis process (which is not performed in the manufacture of CNF) is considered to be a material with high alkali resistance. However, while fibers conventionally used as reinforcing materials have lengths of several tens of millimeters or more, the lengths of NFBC fibers are very short, being several hundred micrometers, so their restraint effect is unclear. It has been reported that the expansion of expansive concrete is suppressed by the mixing of fibers [28], so it is expected that the restraint effect of NFBC can be examined from the viewpoint of the expansion ratio.

From the above, it is thought that NFBC may be used as a new admixture material that will improve the performance of concrete and mortar and have a low impact on the environment. However, there has been almost no research on the use of NFBC in mortar. Therefore, this study used two types of NFBC—a culture solution containing NFBC and a purified solution—to evaluate the influence of NFBC on the basic properties of ordinary and expansive mortars.

## 2. Experimental Outlines

### 2.1. Materials

**Figure 1:**
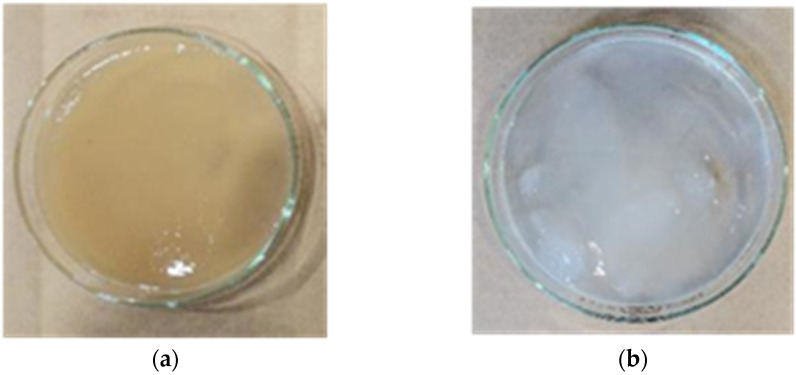
Photographs of the two types of NFBC: (**a**) the culture solution containing NFBC (B_f_); (**b**) the purified solution is obtained by removing bacterial cells and medium components from B_f_ (P_f_).

**Figure 2:**
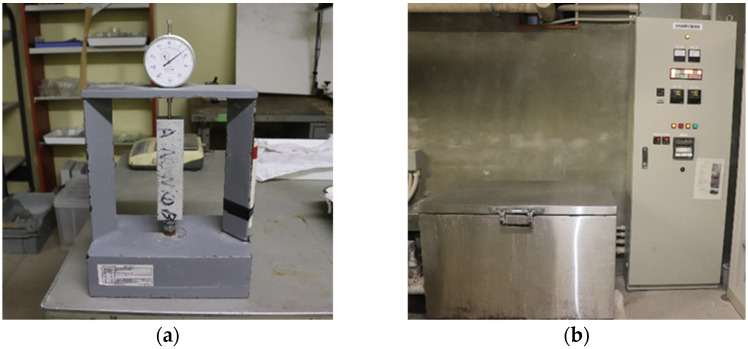
Photographs of the test equipment: (**a**) dial gauge used to measure length-change ratio of dry shrinkage test; (**b**) the test tank of freeze–thaw test.

Table 1 shows the physical properties of the materials. The type of cement used was ordinary Portland cement (OPC), the type of expansive additive (Ex) was ettringite and free CaO, and land sand from Noboribetsu city in Japan was used as the fine aggregate. Figure 1 and Figure 2 show photographs of the two types of NFBC used: a culture solution containing NFBC produced by fermentation from sugar and molasses made from sugar beet and sugar cane (B_f_) and a purified solution obtained by removing bacterial cells and medium components from B_f_ (P_f_). B_f_ and P_f_ were placed in water before mixing in mortar.

### 2.2. Experimental Plan

Table 2 shows the experimental plan. In this research, the binder (b) was OPC and Ex, the water–cement ratio (w/b) was 0.5, the cement–sand ratio (b:s) was 1:3, and the test sample was prepared using B_f_ and P_f_ included as part of the water. From a preliminary experiment, the B_f_ addition ratio was set to 2.5 wt% for cement, which was the maximum addition ratio such that the sample could be demolded 1 day after casting. The P_f_ addition ratio was 0.75 wt% so that the amount of fibers was the same as that of B2.5, 2.5 wt% so that the addition ratio was the same as that of B2.5, and 5.0 wt% so that addition ratio was twice that of P2.5.

### 2.3. Experimental Method

#### 2.3.1. Fresh Properties

The flow and air content were measured according to JIS R 5201 [29] and JIS A 1128 [30], respectively. In the flow test, the flow cone was placed on the flow table was filled with mortar. Then, the flow cone was removed, and 15 falling motions were applied over 15 s to measure the spread of the fresh mortar.

The air content was measured by the method of test for air content of fresh mortar by pressure method using an air content measuring instrument.

The setting time test was conducted in accordance with JIS A 1147 [31]. The setting time was determined from the penetration resistance value of the mortar. The elapsed time from the time of water injection when the penetration resistance values were 3.5 N/mm^2^ and 28.0 N/mm^2^ was defined as the initial setting time and final setting time, respectively.

#### 2.3.2. Restraint Expansion Test

The expansion coefficient under restraint condition was measured according to JCI-S-009-2012 [32] using the φ 100 × 200 mm cylindrical test piece. When the specimens were laid in a room at constant temperature of 20 °C (sealed curing), the expansion was measured from the time of placement to 7 days later using a data logger. The strain gauge was attached to a tin mold, which was measured by a data logger, and the value was used as the restraint expansion coefficient.

#### 2.3.3. Dry Shrinkage Test

The 40 × 40 × 160 mm prism specimens were cured in 20 °C water after being de-molded 1 day after casting for 1 week and were laid in the room at 20 °C and relative humidity (RH) 60%. The mass change ratio and the length change ratio were measured at 7 and 182 days, respectively, after underwater curing. The length-change ratio was measured according to JIS A 1129-3 [33] (see Figure 2a).

#### 2.3.4. Strength Properties

The compressive strength was tested in accordance with JIS A 1108 [34]. The samples were the φ 50 × 100 mm cylindrical test pieces that were cured in 20 °C water after being demolded 1 day after casting (underwater curing) and cured at 20 °C and 60% RH after being demolded 1 day after casting (air curing). The measurements were carried out over 3, 7, and 28 days for underwater curing and over 28 days for air curing.

The splitting tensile strength was tested in accordance with JIS A 1113 [35]. The samples were the φ 50 × 100 mm cylindrical test pieces that were cured in 20 °C water after being demolded 1 day after casting (underwater curing) and those cured at 20 °C and 60% RH after being demolded 1 day after casting (air curing). The measurements were carried out over 28 days for both underwater curing and air curing.

#### 2.3.5. Freeze-Thaw Test

After curing the 40 × 40 × 160 mm prism specimens in 20 °C water for 4 weeks, the temperature was adjusted from −18 °C to +5 °C in cycles over a period of 4 h (see Figure 2b). The relative dynamic modulus of elasticity (RDME), the length-change ratio and the mass-reduction ratio were measured according to A method of JIS A 1148 [36].

## 3. Results and Discussion

### 3.1. Flow and Air Content

**Figure 3:**
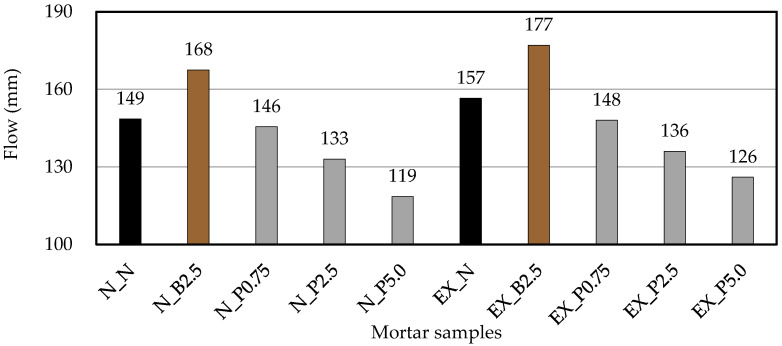
Flow in the fresh mortar. (black: without NFBC, brown: with NFBC type B_f_, grey: with NFBC type P_f_).

Figure 3 shows the flow in the fresh mortar. In comparison to N_N, when B_f_ was added to the mortar, the flow increased, and when P_f_ was added to the mortar, the flow decreased as the addition ratio increased. This may be because the cement paste was restrained by the fibers in P_f_. In previous studies, it was reported that the fluidity decreased as a result of mixing of short polypropylene fibers (PP) and steel fibers [23,37,38], and, similar to other fibers, the fluidity was also decreased by adding P_f_ (Figure 3). It was confirmed that the cause of the increased flow by adding B_f_ is that it contains impurities such as bacterial cells and medium components, but the mechanism could not be clarified in detail in this study. Furthermore, the flow was slightly increased by adding Ex, but, in the case of ordinary mortar, the effect of NFBC was the same as in the case of expansive mortar.

**Figure 4:**
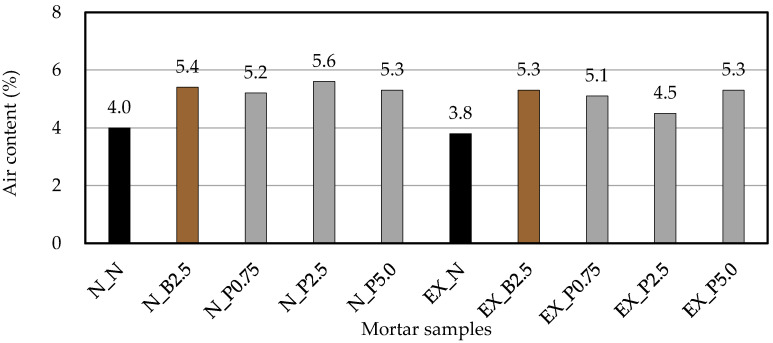
Air content in the fresh mortar. (black: without NFBC, brown: with NFBC type B_f_, grey: with NFBC type P_f_).

Figure 4 shows the air content in the fresh mortar. In this study, comparison is made with mortar that did not use a chemical admixture. Neville [39] states that the air content in concrete depending on the slump and the maximum size of aggregate, and the air content may change on the mix proportion and materials used. Moreover, Peter et al. [40] show that the air content in the mortar depends on the binder used, and it ranges from 4 to 7%. In comparison with N_N, adding Bf and P_f_ increased the air content. However, varying the amount of P_f_ did not significantly change the air content. In case of expansive mortar, the air content increases by B_f_ and P_f_ as compared with EX_N. NFBC may have an air entraining that make the air content of the mortar increase. The air entraining is the same regardless of the amount of NFBC, whether it is added in a small amount (N_B2.5, N_P0.75, EX_B2.5, EX_P0.75) or in a large amount (N_P5.0, EX_P5.0).

### 3.2. Setting Time

**Figure 5:**
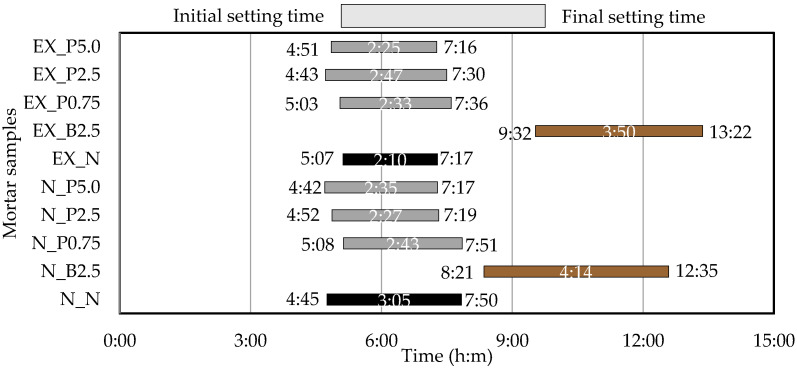
Setting times in ordinary and expansive mortars. (black: without NFBC, brown: with NFBC type B_f_, grey: with NFBC type P_f_).

Figure 5 shows the setting time in the mortar. As compared with N_N and EX_N, the initial setting time and the final setting time were delayed by adding Bf, and the duration was lengthened. This is considered to be the effect of the sugar contained in Bf (contained in the medium components) because sugar inhibits the hydration reaction. In contrast, as compared with N_N, the initial setting time was not significantly affected by adding P_f_, but the duration was slightly shorter, and the final setting time of N_P2.5 and N_P5.0 was approximately 30 min earlier than for N_N. Therefore, compared to EX_N, the addition of P_f_ tends to delay the final setting time slightly and the duration tends to be slightly longer. In previous studies [12], it has been reported that the setting time of mortar is promoted as the CNF made from pulp, and the setting time may be affected by the CNF. However, it is considered that the addition of P_f_ has a smaller effect on the setting time than B_f_.

### 3.3. Expansion Coefficient under Restraint Condition

**Figure 6:**
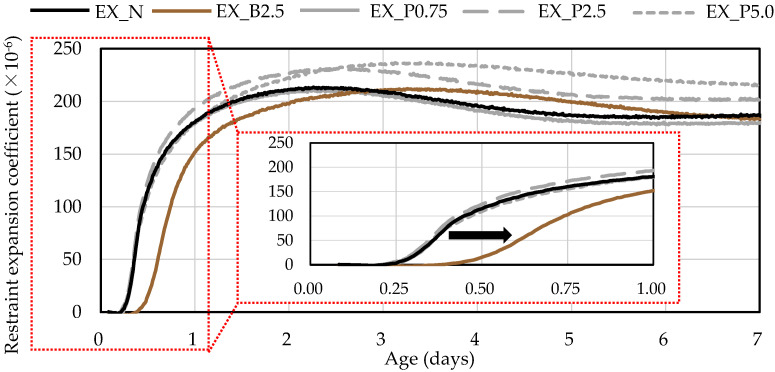
Expansion coefficient under restraint condition in expansive mortars.

Figure 6 shows the expansion coefficient under restraint condition. In EX_N, the restraint expansion coefficient at 7 days was approximately 190 × 10^−6^. This meets the standard set by the Architectural Institute of Japan for a restraint expansion coefficient of 150 × 10^−6^ or greater at 7 days when using Ex [41]. As compared with EX_N, with B_f_, the restraint expansion coefficient was almost unchanged, but the expansion start time was delayed. This is considered to result from the delay in the setting time because sugar inhibits the hydration reaction. From this, it is inferred that the hydration reaction of Ex, as well as that of cement, is delayed by adding B_f_.

On the other hand, there was almost no difference in the restraint expansion coefficient and the expansion start time for EX_P0.75 compared to EX_N, while the restraint expansion coefficient increased for EX_P2.5 and EX_P5.0. This is thought to be because P_f_ reacted with cement to produce an expansive substance or, when superabsorbent polymer was mixed in concrete, internal water curing from P_f_ occurred because the concrete expanded when water was supplied [17,42]. Furthermore, we aimed to clarify the degree of restraint by NFBC, but, in this experiment, it was not possible to confirm the suppression of the restraint expansion coefficient by NFBC. There no expansion was observed and results for the restraint expansion coefficient in N_N, N_B2.5, N_P0.75, N_P2.5, and N_P5.0 were not obtained.

### 3.4. Dry Shrinkage

**Figure 7:**
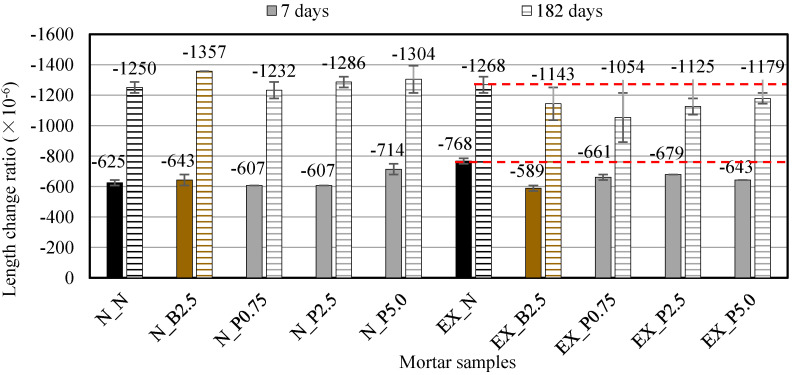
Length-change ratio in the dry environment for ordinary and expansive mortars. (black: without NFBC, brown: with NFBC type B_f_, grey: with NFBC type P_f_).

Figure 7 shows the length-change ratio in the dry environment. As compared with N_N, the length-change ratio was almost unchanged by adding NFBC. On the other hand, as compared with EX_N, the length-change ratio was slightly decreased by adding NFBC.

It is generally accepted that the void structure and shrinkage are closely related. Previous research [43] reported that the shrinkage decreased due to the air entraining admixture, and the shrinkage decreased with the increase in air content. In this study, the air content of the fresh mortar increased by NFBC in both ordinary and expansive mortars. However, the length-change ratio was almost unchanged by adding NFBC to ordinary mortars and the length-change ratio was slightly decreased by adding NFBC to expansive mortars. From these facts, the influence of the air content change by NFBC may be small in the dry shrinkage. Thus, it is considered that the reason that the drying shrinkage of expansive mortars decreased by NFBC is related to the expansion caused by the Ex. Since CNF has high water-retention value [1,4], it is possible that the addition of NFBC caused a hydration reaction of the unreacted Ex even during the drying period, and the expansion might reduce the shrinkage.

From the above, the effect of NFBC on the dry shrinkage of mortar may differ depending on the type of binder.

### 3.5. Compression Strength and Splitting Tensile Strength

**Figure 8:**
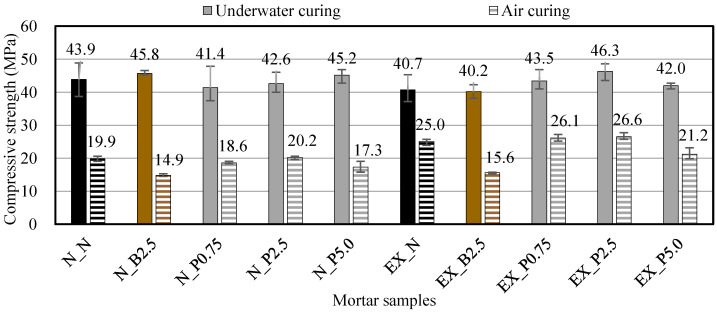
Compressive strengths of ordinary and expansive mortars at 28 days in the different curing conditions. (black: without NFBC, brown: with NFBC type B_f_, grey: with NFBC type P_f_).

Figure 8 shows the compressive strengths of ordinary and expansive mortars after 28 days in the different curing conditions. The compressive strength in air curing decreased compared to that of underwater curing because the hydration reaction did not proceed sufficiently. After adding B_f_, the compressive strength was almost unchanged in the case of underwater curing as compared with N_N and EX_N, but, in the case of air curing, it was slightly decreased. The reduced compressive strength after adding B_f_ is considered to be due to the effect of the sugar contained in B_f_, as previous studies have reported that sugar affects strength [44,45,46]. When P_f_ was added to ordinary mortar, the compressive strength tended to increase slightly as the P_f_ addition ratio increased in the case of underwater curing, but in the case of air curing, the compressive strength increased slightly as the P_f_ addition ratio increased for N_P0.75 and N_P2.5, but did not increase for N_P5.0. Therefore, it is possible that the addition of P_f_ has a different effect on the compressive strength depending on the curing conditions. However, it was confirmed that the compressive strength was not significantly affected by B_f_ and P_f_ in ordinary mortar, because the difference between them was only a few percent as compared with N_N. When P_f_ was added to expansive mortar, the compressive strength increased slightly in underwater curing.

In particular, in EX_P2.5, the compressive strength increased by approximately 14% compared to EX_N. This is considered to be due to the restraint of the fibers in P_f_, because expansive mortar improves the compressive strength due to the restraint [47,48,49]. However, in underwater curing, the compressive strength of EX_P5.0 decreased as compared with EX_P2.5, and in air curing, the compressive strength of EX_P5.0 decreased as compared with EX_N. This indicates that, if the amount of P_f_ is large, it may become an impurity that decreases the strength. From the above, it is considered that the effect of NFBC on the expansive mortar may differ depending on the P_f_ addition ratio and the curing conditions. Then, in this study, in the expansive mortar, the compressive strength of the sample with a P_f_ addition ratio of 2.5 wt% was the largest in the underwater curing case.

**Figure 9:**
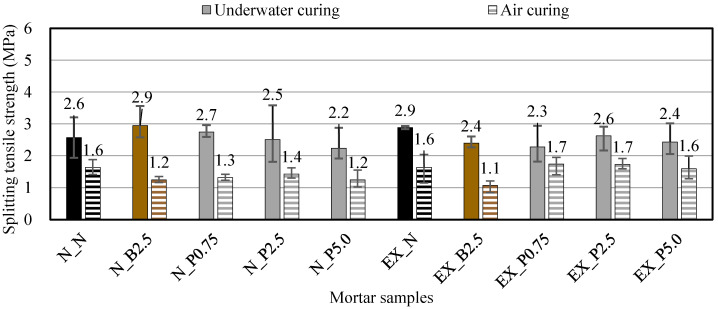
Splitting tensile strengths of ordinary and expansive mortars at 28 days in the different curing conditions. (black: without NFBC, brown: with NFBC type B_f_, grey: with NFBC type P_f_).

Figure 9 shows the splitting tensile strengths of ordinary and expansive mortars at 28 days in the different curing conditions. The splitting tensile strength in air curing decreased more than that of underwater curing also because the hydration reaction did not proceed sufficiently. In underwater curing, the splitting tensile strength slightly increased at N_B2.5 and N_P0.75, and slightly decreased as the P_f_ addition ratio increased, as compared with N_N. In air curing, the splitting tensile strength was not significantly affected by B_f_ and P_f_ as compared with N_N. On the other hand, in underwater curing, the splitting tensile strength slightly decreased for B_f_ and P_f_, as compared with EX_N. In underwater curing, the splitting tensile strength of EX_B2.5 slightly decreased and the splitting tensile strength was not significantly affected by P_f_. It appears that B_f_ and P_f_ do not change the splitting tensile strength much because the difference between them was very small for both ordinary and expansive mortars. The previous study [14] has reported that the CNF made from pulp may improve crack resistance and thus improve the tensile strength of mortar in a dry environment. Therefore, it was expected that NFBC, which is a CNF produced by fermentation using sugar and molasses as raw materials, would also improve the splitting tensile strength of mortar in a dry environment, but this was not obtained in this study.

### 3.6. Frost Resistance

**Figure 10:**
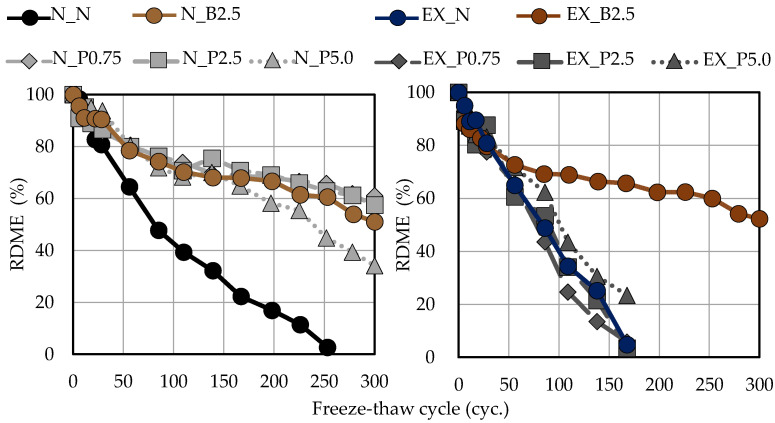
Relationship between RDME and the freeze-thaw cycle in ordinary and expansive mortars. (black: without NFBC, brown: with NFBC type B_f_, grey: with NFBC type P_f_).

Figure 10 shows the relationship between RDME and the freeze-thaw cycle in or-dinary and expansive mortars. The RDME of N_N was less than 60% at around 50 cycles. However, when B_f_ and P_f_ were added to ordinary mortar, RDME was more than 60% at 150 cycles. The reason the frost resistance was improved by adding B_f_ and P_f_ is related to the increased air content as compared to N_N. Furthermore, in previous studies, the frost resistance was improved by adding fibers [50,51,52,53]. Therefore, it was expected that the frost resistance would be improved by the cross-linking effect of the fibers, even if P_f_ was used. However, even if the P_f_ addition ratio in ordinary mortar was increased, the frost resistance did not improve. In addition, RDME of EX_N was less than 60% at around 50 cycles. By adding P_f_, RDME did not change much compared with EX_N, even though the air content increased. RDME of EX_B2.5 was more than 60% at around 250 cycles.

**Figure 11:**
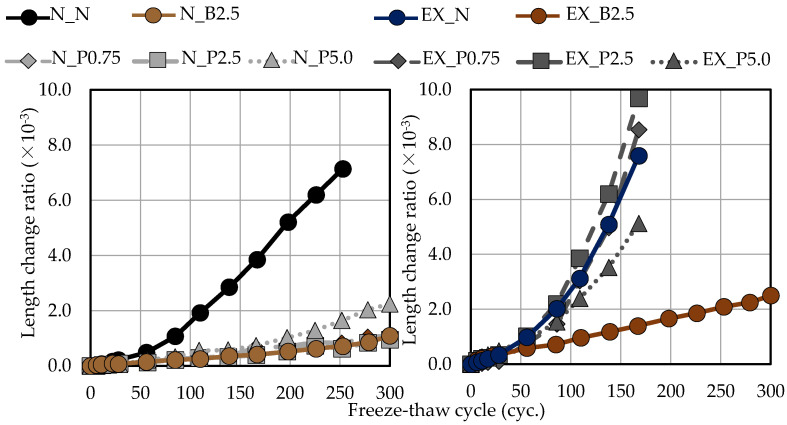
Relationship between length change ratio and the freeze-thaw cycle in ordinary and expansive mortars. (black: without NFBC, brown: with NFBC type B_f_, grey: with NFBC type P_f_).

**Figure 12:**
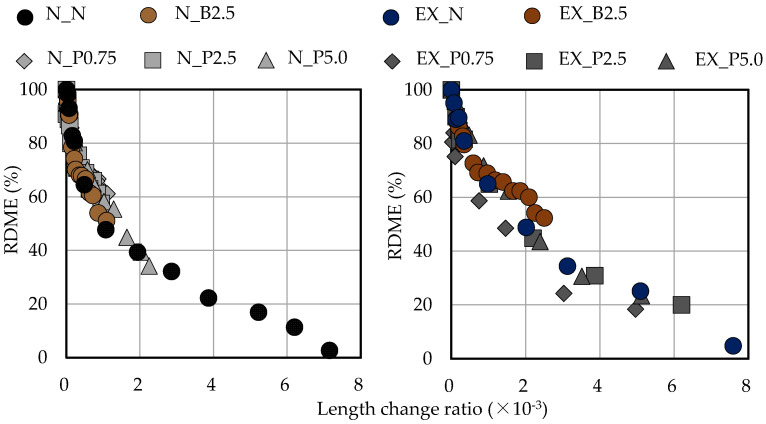
Relationship between RDME and length-change ratio in ordinary and expansive mortars. (black: without NFBC, brown: with NFBC type B_f_, grey: with NFBC type P_f_).

Figure 11 shows the relationship between the length-change ratio and the freeze–thaw cycle in ordinary and expansive mortars. The increase in the length-change ratio was suppressed by B_f_ and P_f_ in ordinary mortar. On the other hand, in expansive mortar, the increase in the length-change ratio was not greatly suppressed by P_f_, while the increase in the length-change ratio was suppressed by Bf. Moreover, when P_f_ was added to expansive mortar, the increase in the length-change ratio was suppressed in EX_P5.0 (the test piece with the highest addition ratio) as compared to EX_N. This may be because of the cross-linking effect of NFBC. In general, it is clear that RDME and the length-change ratio during the freeze–thaw cycle are closely related. For an RDME of 60%, Eiji et al. [54] reported that the length-change ratio was 1.0 × 10^−3^ and Takehumi [55] reported that it was 1.2 × 10^−3^. In this study, as in the previous studies, in ordinary mortar (even when B_f_ and P_f_ were used) when RDME was 60%, the length-change ratio was approximately 1.0 × 10^−3^ (see Figure 12). In the case of expansive mortar, the length-change ratio was larger when RDME was 60% than in ordinary mortar (see Figure 12). It is thought that this is due to the expansion of the unreacted Ex during the freeze-thaw cycle.

**Figure 13:**
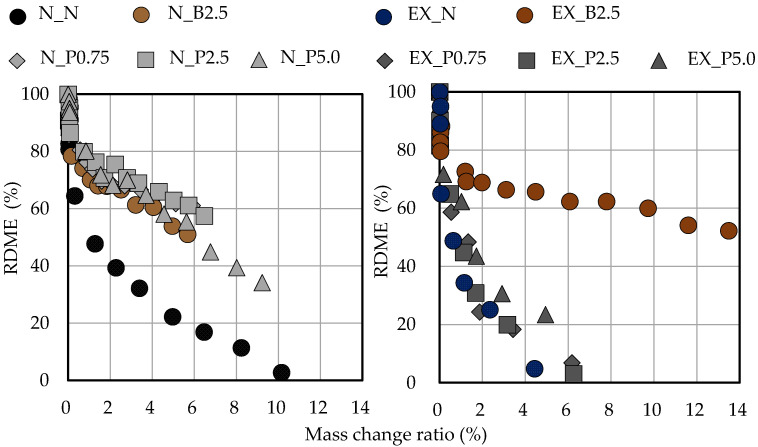
Relationship between RDME and the mass-reduction ratio in ordinary and expansive mortars. (black: without NFBC, brown: with NFBC type B_f_, grey: with NFBC type P_f_).

Figure 13 shows the relationship between RDME and the mass-reduction ratio in the ordinary and expansive mortars. When B_f_ and P_f_ were added to ordinary mortar, RDME was less likely to decrease even if the mass-reduction ratio increased, as compared with N_N. Furthermore, when B_f_ was added to expansive mortar, RDME was less likely to decrease even if the mass-reduction ratio increased, as compared with N_N. On the other hand, the relationship between RDME and the mass-reduction ratio when P_f_ was added to expansive mortar was similar to that of EX_N. Therefore, the addition of B_f_ and P_f_ may change the deterioration due to freeze-thaw cycles.

From the above, it is possible that the effect of NFBC on mortar differs depending on the type of binder. The frost resistance was improved by adding B_f_, regardless of the binder, and by adding P_f_ in ordinary mortar.

## 4. Conclusions

This paper aims to investigate the influence of NFBC on the basic properties of ordinary and expansive mortar. As a result, the flow of mortar increases with the addition of the culture solution containing NFBC is produced by fermentation from sugar and molasses made from sugar beet and sugar cane (B_f_) and decreases with the increase of the purified solution is obtained by removing bacterial cells and medium components from B_f_ (P_f_) addition ratio. Meanwhile, the air content increases with the addition of B_f_ and P_f_. NFBC may have an air entrainment, and as a result, the air content of mortar increases. By adding B_f_, the initial setting time and the final setting time are delayed and duration increased. It is clear that the addition of P_f_ has a smaller effect on the setting time than B_f_. In addition, the compressive strength and splitting tensile strength at 28 days are not significantly affected by adding B_f_ and P_f_ in the case of ordinary mortar, regardless of the curing conditions. In expansive mortar with P_f_, the compressive strength increases slightly at 28 days of underwater curing. The dry shrinkage is slightly decreased by adding NFBC in expansive mortar. The frost resistance is improved by adding B_f_ and P_f_ in the case of ordinary mortar. It is considered that this is because the air volume in the fresh mortar increases by adding B_f_ and P_f_. In the case of expansive mortar, the frost resistance is improved by adding B_f_, but not improved by adding P_f_.

Overall, it was confirmed that these types of nanofibrillated bacterial cellulose (NFBC) can be used as admixtures to improve the performance of cementitious materials, such as freezing resistance. However, these mechanisms were not elucidated in detail in this study, and should be left as a subject for future investigation. In addition, the microstructure of NFBC mortar will be clarified by mercury injection porosimetry (MIP) test and SEM analysis.

## Figures and Tables

**Table 1:** Physical properties of the materials.

Material	Symbol	Classification and Physical Properties
Cement	OPC	Ordinary Portland cement Specific gravity: 3.17 g/cm^3^
Expansive additive	Ex	Ettringite and free CaO Specific gravity: 3.17 g/cm^3^ Standard usage: 20 kg/m^3^
Sand	S	Land sand from Noboribetsu city in Japan Saturated density: 2.73 g/cm^3^ Oven-dry density: 2.68 g/cm^3^ Absorption: 1.72%
Nanofibrillated Bacterial Cellulose (NFBC)	B_f_	CNF produced by fermentation culture solution containing bacterial cells and medium components in addition to fibers Fiber ratio: ~0.3 wt% Fiber width: 10–50 nm Fiber length: 100–500 µm
	P_f_	Purified product from B_f_ with bacterial cells and medium components removed Fiber ratio: ~1.0 wt% Fiber width: 10–50 nm Fiber length: 100–500 µm

**Table 2:** Experimental plan.

Symbol	w/b	b (%)		NFBC	B_f_ or P_f_ Ratio b × wt (%)	Fiber Ratio in B_f_ or P_f_ b × wt (%)
		OPC	Ex			
N_N	0.5	100	0	-	0	0
N_B2.5				B_f_	2.5	0.0075
N_P0.75				P_f_	0.75	
N_P2.5					2.5	0.025
N_P5.0					5.0	0.05
EX_N		95	5	-	0	0
EX_B2.5				B_f_	2.5	0.0075
EX_P0.75				P_f_	0.75	
EX_P2.5					2.5	0.025
EX_P5.0					5.0	0.05

## Data Availability

Not applicable.
